# Supplementary material for: Wildfire imagery reduces risk information-seeking among homeowners as property wildfire risk increases
Source: Commun Earth Environ. 2022 Oct 4;3(1):229. doi: 10.1038/s43247-022-00505-7 (PMC9531637; doi:10.1038/s43247-022-00505-7)
Supplement: Supplementary file 1 — Supplementary Information [file 43247_2022_505_MOESM1_ESM.pdf]

Supplementary Information for *Wildfire imagery reduces risk information-seeking among homeowners as property wildfire risk increases*

Supplementary Table 1. Descriptive statistics and balance across treatments for property owners who received postcards in the field experiment.

	**Overall**<br>n = 5,785	**Flames**<br>n = 2,893	**Status Quo**<br>n = 2,892
**Risk score**	443 (127)	443 (126)	444 (127)
**Property size (acres)**	0.26 (0.36)	0.26 (0.30)	0.26 (0.40)
**Property value ($k)**	488 (201)	486 (178)	490 (221)
**Year built**	1968 (33)	1967 (33)*	1969 (32)*
**Multiple properties**[a]	454 (7.8%)	220 (7.6%)	234 (8.1%)
**Part-time resident**[b]	953 (16%)	505 (17%)*	448 (15%)*

Note: Mean (SD); number (%). Property value is in thousands of US dollars. [A]Owner has more than one property in the city. [B]Owner's mailing address is outside of the city. Differences between the Flames and Status Quo groups were tested using Wilcoxon rank sum and Pearson's Chi-squared tests. *$p < 0.05$

Supplementary Table 2. Online experiment participant responses to the two photos used in the field experiment (Figure 1).

Measure	Status Quo (n = 111)	Flames (n = 109)	Statistic
Emotional Responses (The photo makes me feel…)			
Worried	5.3 (3)	8 (2)	-7.4***
Anxious	4.6 (3)	7 (2)	-7***
Fearful	4.9 (3)	7 (2)	-5.8***
Calm	3.6 (3)	2 (2)	5.5***
Peaceful	3.6 (3)	1 (2)	6.5***
Safe	3.6 (3)	1 (2)	7.3***
Risk Assessments			
Chance of fire (%)	45 (30)	50 (30)	-1.2
Chance of fire damage (%)	60 (20)	60 (30)	-0.7
How risky is living here	6.2 (2)	7 (2)	-2.6*
Behavioral Intentions			
Receive more information	4.4 (0.8)	5 (0.8)	-0.8
Visit personalized website	3.7 (0.6)	4 (0.5)	-0.2
Personal Preferences			
Like the photo	7.6 (2)	5 (3)	8.4***
Photo is personally relevant	5.6 (3)	4 (3)	4.3***

Note: Columns 2-3 show Mean (SD). Statistics are from two-sided t-tests. Measures of emotion and personal reactions were rated on a Likert scale from 'Not at all' (0) to 'The most possible' (10). Responses to 'Chance of…' questions were rated from 'No chance' (0%) to 'For sure' (100%); 'How risky is living here' was rated from 0 = 'Not risky at all' to 10 = 'Extremely risky'. Behavioral intentions were rated on a 5-point Likert scale from 'Extremely unlikely' (1) to 'Extremely likely' (5). P-values are adjusted for multiple comparisons using the Benjamini-Hochberg method: * $p < 0.05$; *** $p < 0.001$

Supplementary Table 3. Online experiment participant responses to the four photos (Supplementary Figure 1).

	**Flames** *B1* (n = 109)	**Flames** *B2* (n = 111)	**Status Quo** *A1* (n = 111)	**Status Quo** *A2* (n = 109)	$F_{(3,432+)}$
Emotional Responses (The photo makes me feel…)					
Worried	6.9 (2)	7.0 (2)	4.6 (3)	4.5 (3)	35.5***
Anxious	6.8 (2)	6.9 (2)	4.9 (3)	4.4 (3)	30.3***
Fearful	7.6 (2)	7.6 (2)	5.3 (3)	4.8 (3)	45.1***
Calm	1.8 (2)	1.4 (2)	3.6 (3)	4.3 (3)	37.2***
Peaceful	1.5 (2)	1.0 (2)	3.6 (3)	4.3 (3)	48.0***
Safe	1.4 (2)	1.2 (2)	3.6 (3)	4.0 (3)	46.8***
Risk Assessments					
Chance of fire (%)	49 (30)	50 (30)	45 (30)	46 (30)	0.864
Chance of fire damage (%)	62 (30)	61 (20)	60 (20)	60 (20)	0.233
How risky is living here	7.0 (2)	7.3 (2)	6.2 (2)	6.0 (2)	9.05***
Behavioral Intentions					
Receive more information	4.5 (0.8)	4.6 (0.7)	4.4 (0.8)	4.4 (0.8)	1.86
Visit personalized website	3.7 (0.5)	3.8 (0.5)	3.7 (0.6)	3.7 (0.6)	1.14
Personal Preferences					
Like the photo	4.6 (3)	3.4 (3)	7.6 (2)	7.4 (2)	71.9***
Photo is personally relevant	3.9 (3)	4.0 (3)	5.6 (3)	5.3 (3)	10.4***

Note: Columns 2-5 show Mean (SD) responses to each measure. F-statistics are from one-way ANOVAs with a minimum n = 432 (some measures had missing observations). Measures of emotion and personal reactions were rated on a Likert scale from 'Not at all' (0) to 'The most possible' (10). Responses to 'Chance of…' questions were rated from 'No chance' (0%) to 'For sure' (100%); 'How risky is living here' was rated from 0 = 'Not risky at all' to 10 = 'Extremely risky'. Behavioral intentions were rated on a 5-point Likert scale from 'Extremely unlikely' (1) to 'Extremely likely' (5). * $p < 0.05$; *** $p < 0.001$

Supplementary Table 4. Descriptive statistics of homeowners who visited their risk webpages in the field experiment, overall and by treatment group.

	**Overall**<br>n = 1,116	**Flames**<br>n = 545	**Status Quo**<br>n = 571
**Risk score**	475 (111)	466 (114)	483 (109)
**Property size (acres)**	0.27 (0.30)	0.26 (0.22)	0.28 (0.37)
**Property value ($k)**	496 (171)	498 (182)	495 (160)
**Year built**	1971 (30)	1969 (31)	1972 (29)
**Multiple properties[a]**	81 (7.3%)	40 (7.3%)	41 (7.2%)
**Part-time resident[b]**	105 (9.4%)	51 (9.4%)	54 (9.5%)
**Visited proportion[c]**	19.3%	18.8%	19.7%

Note: Mean (SD); number (%). Property value is in thousands of US dollars. [a]Owner has more than one property in the city. [b]Owner's mailing address is outside of the city. [c]Calculated from number of homeowners in each sample (Overall = 5785; Flames = 2893; Status Quo = 2892).

Supplementary Figure 1. Four photos used in the online experiment.

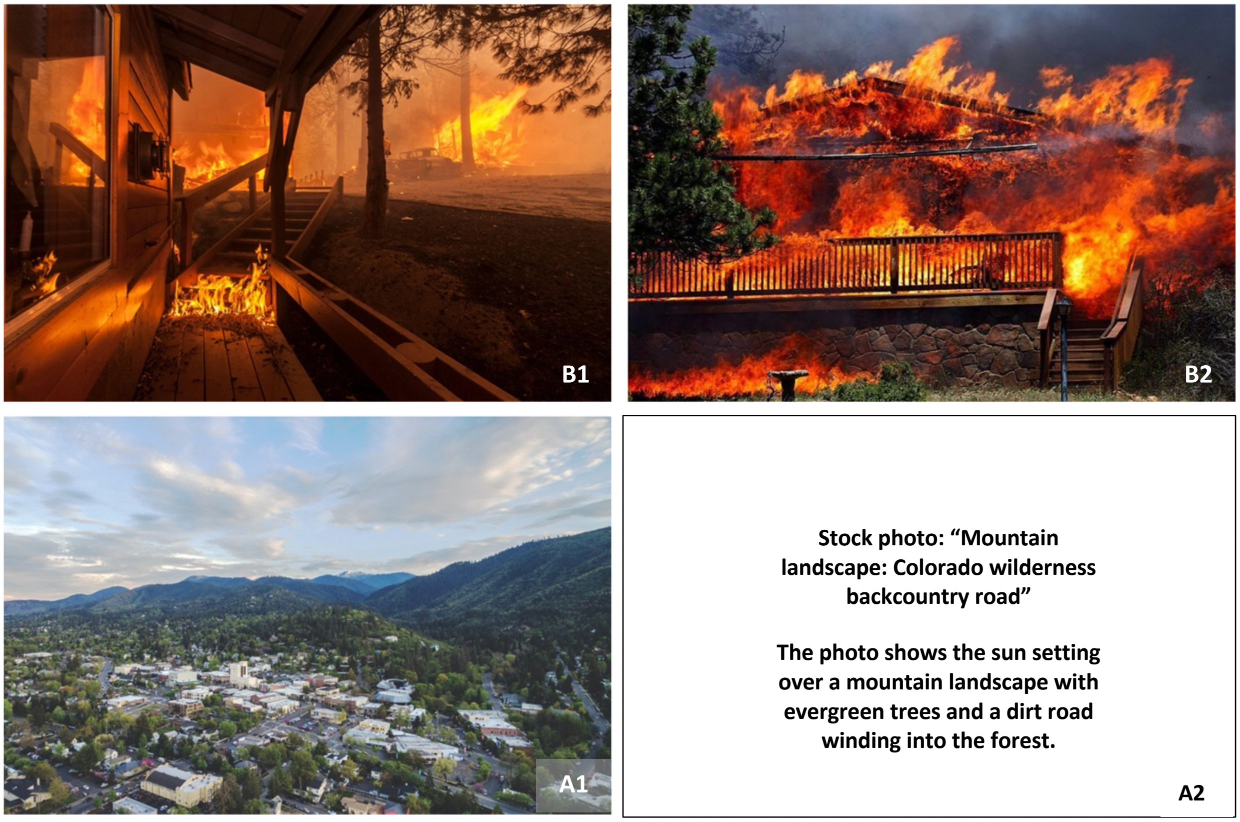

Note: Participants in were randomly assigned to see one of these four photos. Two photos (top) appeared in media outlets describing wildfire. Two photos (bottom) have been used in wildfire communications to homeowners from local fire departments. The two photos on the left (B1 & A1) were also used in the wildfire practitioner survey and field experiment with homeowners. Photo credits: Mark Thiessen (top left), Darrell Spangler (top right), Darren Campbell (bottom left). Contact corresponding author to view the A2 photo.

Supplementary Figure 2. Screenshot of the top portion of a property-specific wildfire risk webpage. The property shown is Ashland Fire and Rescue.

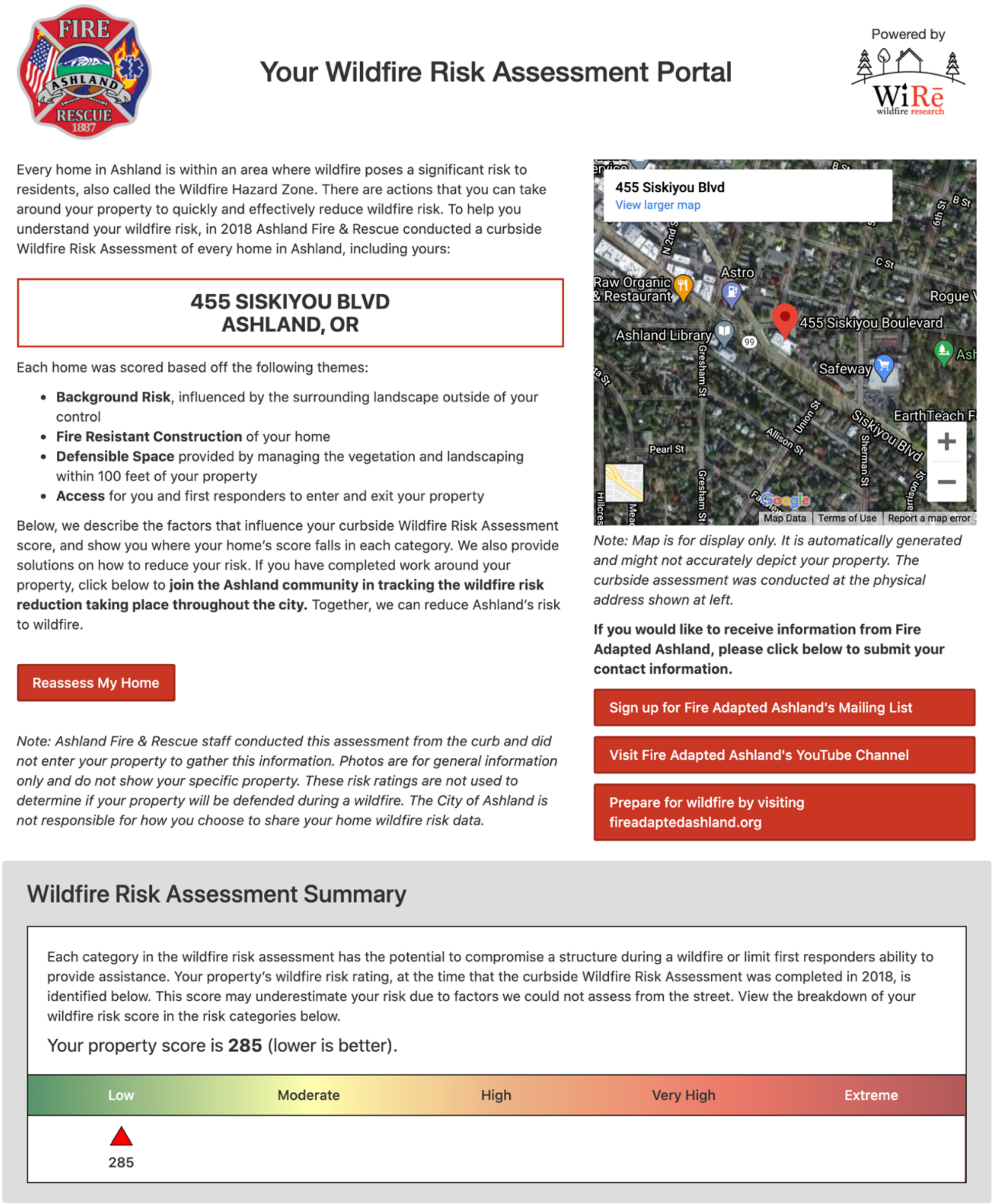

Note: Scrolling down, the visitor would see the breakdown of their property's risk factors, including background risk, construction, defensible space, and emergency access. Map data ©2020 Google.

Supplementary Figure 3. Backside of a postcard mailed to homeowners.

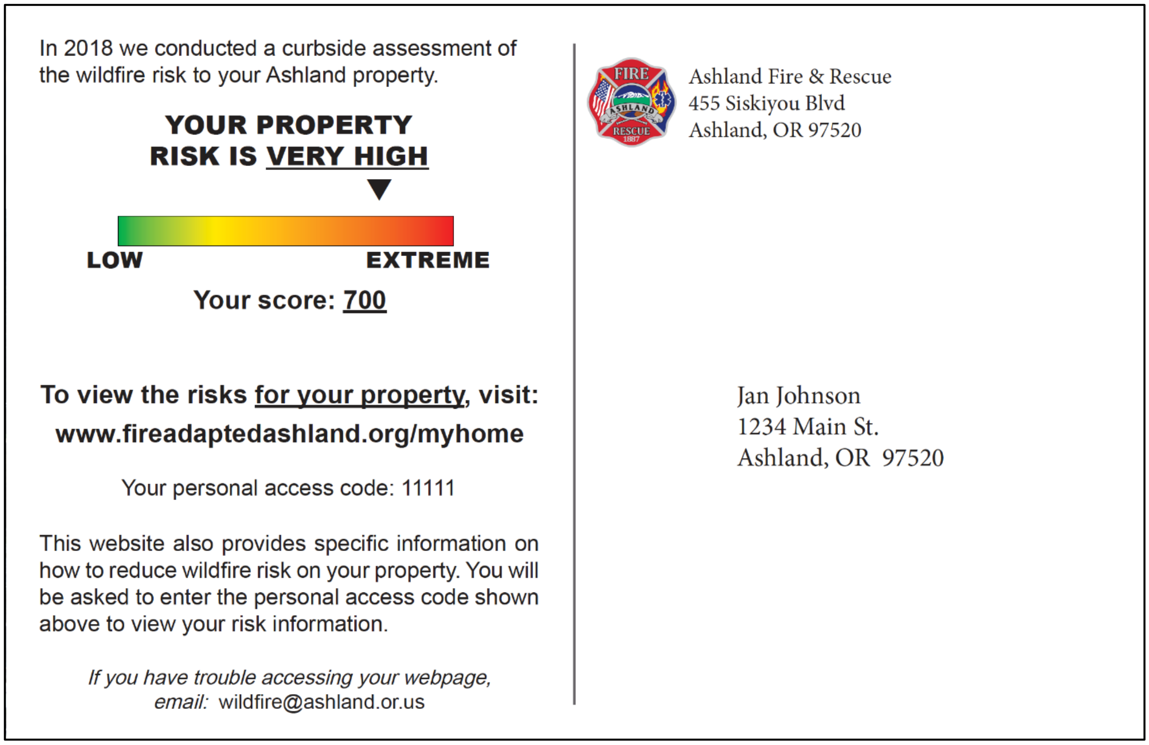

In 2018 we conducted a curbside assessment of the wildfire risk to your Ashland property.

YOUR PROPERTY RISK IS VERY HIGH

LOW **EXTREME**

Your score: 700

To view the risks for your property, visit:
www.fireadaptedashland.org/myhome

Your personal access code: 11111

This website also provides specific information on how to reduce wildfire risk on your property. You will be asked to enter the personal access code shown above to view your risk information.

If you have trouble accessing your webpage, email: wildfire@ashland.or.us

Ashland Fire & Rescue
455 Siskiyou Blvd
Ashland, OR 97520

Jan Johnson
1234 Main St.
Ashland, OR 97520

Note: The property risk rating, risk score, personal access code, and addressee were tailored to the recipient homeowner.

Supplementary Figure 4. Differences in responses to the four photos in the online experiment.

Anxious

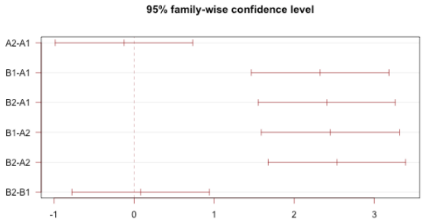

Fearful

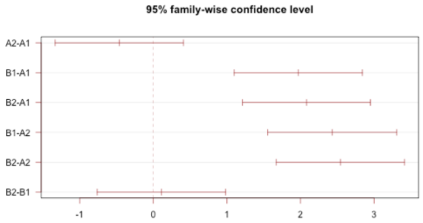

Worried

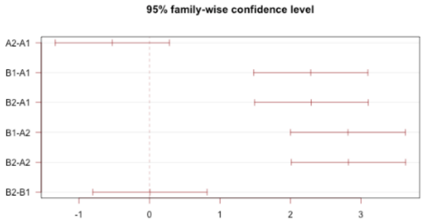

Calm

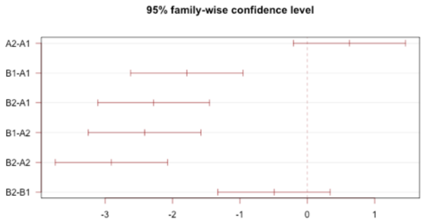

Peaceful

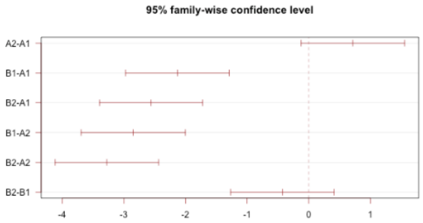

Safe

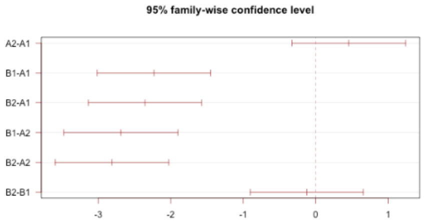

Chance of fire

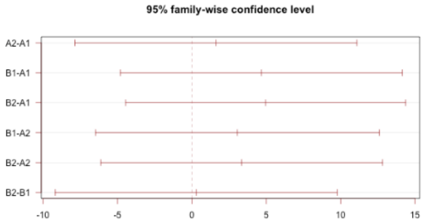

Chance of damaging home

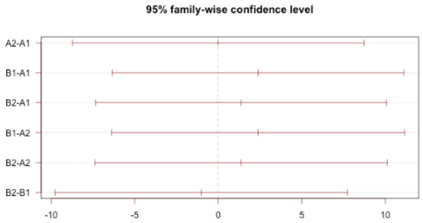

Risk of living here

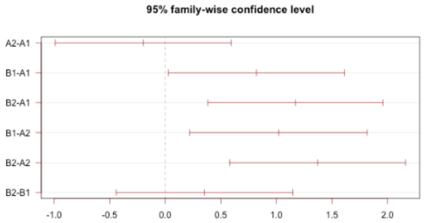

Intention to receive information

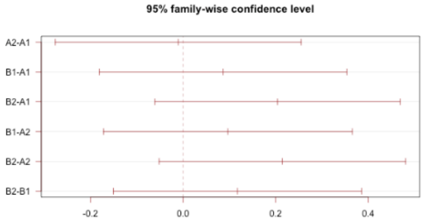

Intention to visit webpage

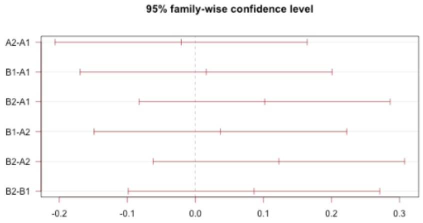

Like photo

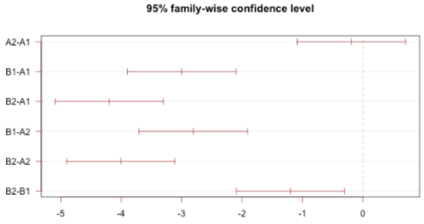

Photo is personally relevant

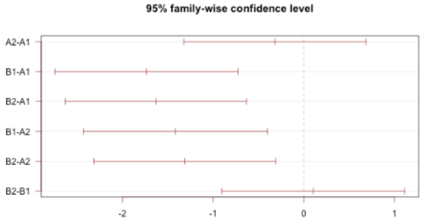

Note: Results are from Tukey's HSD tests. X-axis shows differences in responses from Likert scales. Y-axis shows pairwise comparisons between four photos (Supplementary Figure 1), which include two photos used across the three studies (A1 = status quo; B1 = flames) and two similar photos used in wildfire communications (A2 = status quo-other) and mainstream media (B2 = flames-other).

Supplementary Figure 5. Actual visitation of homeowners to their wildfire risk webpages by parcel-level wildfire risk (x-axis) and the photo on the postcard they received (fill color).

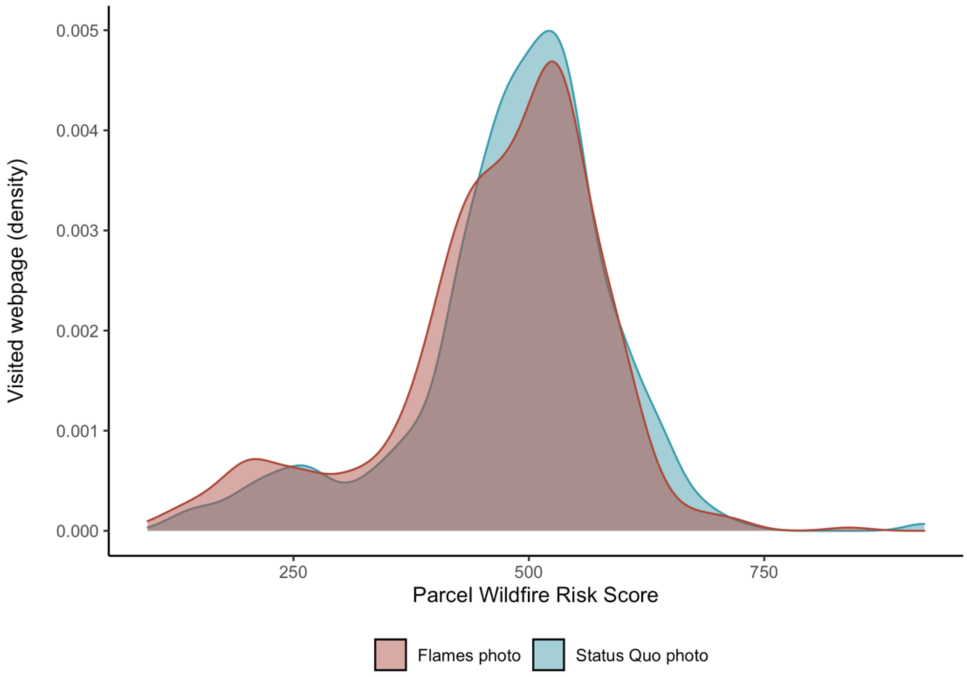

Supplementary Methods

1. Practitioner Survey
2. Online Experiment Survey

1. Practitioner Survey

Please take a moment to study the two photos below.

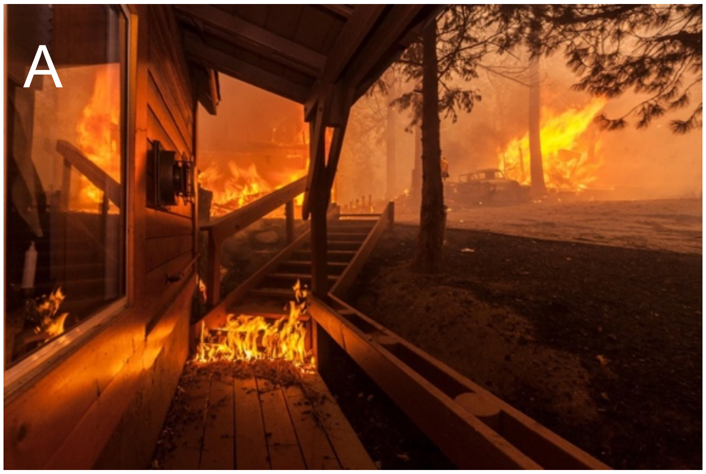

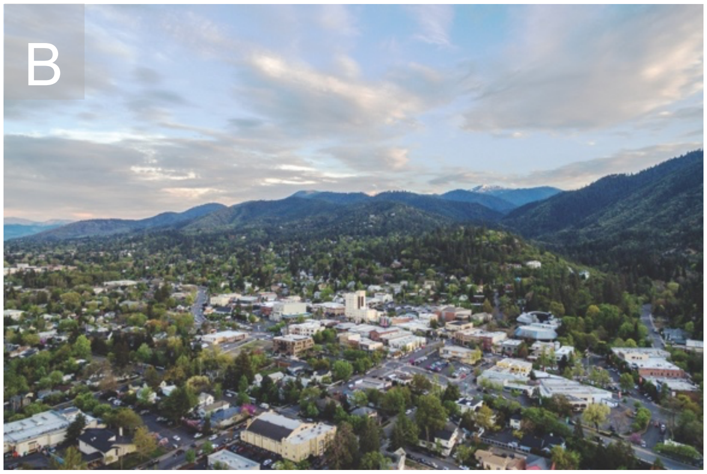

1. Which photo is more effective at engaging homeowners to learn more about their wildfire risk?
   - Photo A (flames)
   - Photo B (landscape)
   - No difference

   1.a. Why? ___________________________

2. Which photo would you choose to use in your outreach communications to homeowners about wildfire risk? For example, on a postcard informing homeowners about their properties' wildfire risk.
   - Photo A (flames)
   - Photo B (landscape)
   - No difference

   2.a. Why? ___________________________

3. In your experience, is there agreement between wildfire practitioners about whether photos of flames (i.e. worst-case scenario) engage or repel homeowners when communicating about wildfire risk?
   - **Yes**, most think flames engage homeowners
   - **Yes**, most think flames repel homeowners
   - **No**, there is not agreement about the effect of photos of flames on homeowner engagement
   - Most practitioners do not think photos matter in communicating with homeowners

2. Online Experiment Survey

Imagine you own a home in an area that is at risk from wildfire. *There have been 10 large wildfires in the area in the last 100 years.*

You receive a postcard in the mail from the local fire department. On one side, you are informed that your property is HIGH RISK for wildfire. On the other side, you see the image below.

PHOTO[*]

To what extent does this photo make you feel…

	**None**									**Extremely**
Worried	0	1	2	3	4	5	6	7	8	9
Calm	0	1	2	3	4	5	6	7	8	9
Anxious	0	1	2	3	4	5	6	7	8	9
Fearful	0	1	2	3	4	5	6	7	8	9
Peaceful	0	1	2	3	4	5	6	7	8	9
Safe	0	1	2	3	4	5	6	7	8	9

[*] *participant randomly assigned to view one of four photos*

Imagine you own a home in an area that is at risk from wildfire. *There have been 10 large wildfires in the area in the last 100 years.*

You receive a postcard in the mail from the local fire department. On one side, you are informed that your property is HIGH RISK for wildfire. On the other side, you see the image below.

PHOTO

What do you think is the chance that a wildfire will occur in this area this year?

**No chance**										**For sure**
0%	10%	20%	30%	40%	50%	60%	70%	80%	90%	100%

If there is a wildfire in this area, what do you think is the chance that it would damage your home?

**No chance**										**For sure**
0%	10%	20%	30%	40%	50%	60%	70%	80%	90%	100%

How risky do you consider living in a place like this?

**Not risky at all**										**Extremely risky**
0%	10%	20%	30%	40%	50%	60%	70%	80%	90%	100%

Imagine you own a home in an area that is at risk from wildfire. *There have been 10 large wildfires in the area in the last 100 years.*

You receive a postcard in the mail from the local fire department. On one side, you are informed that your property is HIGH RISK for wildfire. On the other side, you see the image below.

PHOTO

After receiving this postcard, I would be interested in receiving more information about wildfire in my local community.

Strongly disagree	Somewhat disagree	Neither agree nor disagree	Somewhat agree	Strongly agree
O	O	O	O	O

If the local fire department created a website that shows wildfire risk information <u>specific to your property,</u> how likely are you to visit this website?

Extremely unlikely	Somewhat unlikely	Neither likely nor unlikely	Somewhat likely	Extremely likely
O	O	O	O	O

Imagine you own a home in an area that is at risk from wildfire. *There have been 10 large wildfires in the area in the last 100 years.*

You receive a postcard in the mail from the local fire department. On one side, you are informed that your property is HIGH RISK for wildfire. On the other side, you see the image below.

PHOTO

How much do you like this photo?

**Not at all**										**The most possible**
0	1	2	3	4	5	6	7	8	9	10

How personally relevant is this photo?

**Not at all**										**The most possible**
0	1	2	3	4	5	6	7	8	9	10
