# Supplementary material for: Wildfire imagery reduces risk information-seeking among homeowners as property wildfire risk increases
Source: Commun Earth Environ. 2022 Oct 4;3(1):229. doi: 10.1038/s43247-022-00505-7 (PMC9531637; doi:10.1038/s43247-022-00505-7)
Supplement: Supplementary file 3 — Reporting Summary [file 43247_2022_505_MOESM3_ESM.pdf]

nature research

Corresponding author(s): Hilary Byerly Flint
Manuscript COMMSENV-21-0651B

Last updated by author(s): Jul 14, 2022

Reporting Summary

Nature Research wishes to improve the reproducibility of the work that we publish. This form provides structure for consistency and transparency in reporting. For further information on Nature Research policies, see our Editorial Policies and the Editorial Policy Checklist.

Statistics

For all statistical analyses, confirm that the following items are present in the figure legend, table legend, main text, or Methods section.

n/a	Confirmed	
☐	☒	The exact sample size (n) for each experimental group/condition, given as a discrete number and unit of measurement
☐	☒	A statement on whether measurements were taken from distinct samples or whether the same sample was measured repeatedly
☐	☒	The statistical test(s) used AND whether they are one- or two-sided<br>*Only common tests should be described solely by name; describe more complex techniques in the Methods section.*
☐	☒	A description of all covariates tested
☐	☒	A description of any assumptions or corrections, such as tests of normality and adjustment for multiple comparisons
☐	☒	A full description of the statistical parameters including central tendency (e.g. means) or other basic estimates (e.g. regression coefficient) AND variation (e.g. standard deviation) or associated estimates of uncertainty (e.g. confidence intervals)
☐	☒	For null hypothesis testing, the test statistic (e.g. F, t, r) with confidence intervals, effect sizes, degrees of freedom and P value noted<br>*Give P values as exact values whenever suitable.*
☒	☐	For Bayesian analysis, information on the choice of priors and Markov chain Monte Carlo settings
☒	☐	For hierarchical and complex designs, identification of the appropriate level for tests and full reporting of outcomes
☒	☐	Estimates of effect sizes (e.g. Cohen's d, Pearson's r), indicating how they were calculated

Our web collection on statistics for biologists contains articles on many of the points above.

Software and code

Policy information about availability of computer code

Data collection	N/A
Data analysis	R statistical software was used to analyze the data

For manuscripts utilizing custom algorithms or software that are central to the research but not yet described in published literature, software must be made available to editors and reviewers. We strongly encourage code deposition in a community repository (e.g. GitHub). See the Nature Research guidelines for submitting code & software for further information.

Data

Policy information about availability of data

All manuscripts must include a data availability statement. This statement should provide the following information, where applicable:
- Accession codes, unique identifiers, or web links for publicly available datasets
- A list of figures that have associated raw data
- A description of any restrictions on data availability

The data that support the findings of this study will be made available on the project site on Open Science Framework (https://osf.io/fa2ms). The practitioner and online surveys are included in the supplementary information.

Field-specific reporting

Please select the one below that is the best fit for your research. If you are not sure, read the appropriate sections before making your selection.

☐ Life sciences ☒ Behavioural & social sciences ☐ Ecological, evolutionary & environmental sciences

For a reference copy of the document with all sections, see nature.com/documents/nr-reporting-summary-flat.pdf

Behavioural & social sciences study design

All studies must disclose on these points even when the disclosure is negative.

Study description	This is a quantitative study that included two surveys and a field experiment.
Research sample	Study 1 sample: wildfire practitioners who are members of the Fire Adapted Communities Learning Network (https://fireadaptednetwork.org/). This is a national network of wildfire professionals actively working on fire adaptation, including representatives from fire departments, conservation districts, nonprofits, and Firewise fire councils.<br>Study 2: online research participants from Prolific (www.prolific.co) who are self-reported homeowners and reside in one of the 15 most wildfire-prone states in the United States.<br>Study 3:The sample frame included owners of every parcel within the city limits of Ashland, Oregon (N = 6,400).
Sampling strategy	Study 1: convenience sample<br>Study 2: convenience sample<br>Study 3: census<br>Power analyses were conducted for each study to determine the number of participants necessary to detect the selected effect size.
Data collection	Study 1: participants completed an online survey<br>Study 2: participants completed an online survey<br>Study 3: participants were unaware of their participation in the field experiment
Timing	Data collection took place over several months in Summer 2020
Data exclusions	No data were excluded from the analysis.
Non-participation	Given the convenience sampling and broadcasting methods used in Study 1 and Study 2, we do no know the response rates. For Study 3, the outcome measure is the response rate.
Randomization	Study 1: Participants were not allocated into experimental groups<br>Study 2: Participants were randomly assigned into one of treatment groups using the randomization functionality of the survey software, Qualtrics.<br>Study 3: Assignment to treatment followed a randomized block design, in which homeowners were randomly and evenly distributed between the treatment and control groups according to their wildfire risk rating (Low to Extreme), which was classified by wildfire experts using wildfire risk scores.

Reporting for specific materials, systems and methods

We require information from authors about some types of materials, experimental systems and methods used in many studies. Here, indicate whether each material, system or method listed is relevant to your study. If you are not sure if a list item applies to your research, read the appropriate section before selecting a response.

Materials & experimental systems

n/a	Involved in the study	
☒	☐	Antibodies
☒	☐	Eukaryotic cell lines
☒	☐	Palaeontology and archaeology
☒	☐	Animals and other organisms
☐	☒	Human research participants
☒	☐	Clinical data
☒	☐	Dual use research of concern

Methods

n/a	Involved in the study	
☒	☐	ChIP-seq
☒	☐	Flow cytometry
☒	☐	MRI-based neuroimaging

Human research participants

Policy information about studies involving human research participants

Population characteristics	We did not collect demographic information for participants in our studies.

Recruitment	Study 1: Practitioners were recruited through emails to contacts and postings on the Fire Adapted Communities Learning Network (https://fireadaptednetwork.org/) message board. The wildfire professionals who responded to our survey may not represent the broader population of wildfire practitioners. These individuals are part of an online community of fire professionals interested in building community resilience to wildfire. As such, these practitioners may be more informed about and experienced with wildfire outreach to homeowners. It is unclear whether or how their perceptions might differ from the broader population of wildfire practitioners. However, this subpopulation is integral to building fire-adapted communities in the United States.<br>Study 2: Participants were recruited through the online research platform Prolific (www.prolific.co) and compensated $1 for their participation. According to Prolific, "Participants can browse the available studies, reading descriptions, comparing hourly reward rates and average completion time. This means that every participant in your sample has chosen to do your study. Consequently, it is possible that the people who participate in your study differ systematically from the wider population: they may be particularly interested in the topic of your study, or were attracted by the reward's magnitude." (https://researcher-help.prolific.co/hc/en-gb/articles/360009501473-What-are-the-advantages-and-limitations-of-an-online-sample-)<br>Study 3: Participants were recruited through a mailing from the local fire department. The natural setting of this study prevented selection bias.
Ethics oversight	Ethical approval was provided by the University of Colorado, Boulder Institutional Review Board

Note that full information on the approval of the study protocol must also be provided in the manuscript.
